# Clinical and Antibacterial Effects of Tualang Honey on Pseudomonas-induced Keratitis in Rabbit Eyes

**DOI:** 10.7759/cureus.4332

**Published:** 2019-03-27

**Authors:** Rajendran Punitan, Siti Amrah Sulaiman, Habsah B Hasan, Ismail Shatriah

**Affiliations:** 1 Ophthalmology, School of Medical Sciences, Universiti Sains Malaysia, Kubang Kerian, MYS; 2 Pharmacology, School of Medical Sciences, Universiti Sains Malaysia, Kubang Kerian, MYS; 3 Microbiology and Parasitology, School of Medical Sciences, Universiti Sains Malaysia, Kubang Kerian, MYS

**Keywords:** tualang honey, pseudomonas aeruginosa, pseudomonas-induced keratitis

## Abstract

Introduction

Pseudomonas aeruginosa is a common cause of microbial keratitis that can cause a significant loss of visual acuity. Antibiotics, including fluoroquinolones and aminoglycosides, are clinically effective against Pseudomonas-induced keratitis, but their effectiveness has been conspicuously reduced as resistant pathogens have become more potent. This study sought to evaluate the clinical and antibacterial effects of tualang honey as an alternative therapeutic agent against Pseudomonas-induced keratitis.

Methods

We conducted a randomized control trial in which 30 rabbits were injected intrastromally with 1,000 colony-forming units (CFU) of Pseudomonas aeruginosa in the right eye of each rabbit (n = 30). The rabbits were then randomized into three groups of 10 rabbits each. Group A was treated with topical gentamicin 0.3%, group B was treated with topical tualang honey 30%, and group C received both treatments. The specified treatments were administered every two hours from 24 to 48 hours post injection, and subsequently every four hours for six days. Clinical examinations were performed on days one, two, three, five, and seven, and the mean results of slit lamp examinations (SLEs) were documented. On day seven after pseudomonas induction, the rabbits were euthanized and their corneas were harvested to determine the median CFU per cornea.

Results

There were no statistically significant differences (p > 0.05) in mean SLE scores (p = 0.209) or median CFU values (p = 0.820) between the three groups.

Conclusion

Topical gentamicin, topical tualang honey, and the combination of the two all showed similar clinical and antimicrobial effects in treating Pseudomonas-induced keratitis in rabbits. These findings should be verified in further studies with larger sample sizes and the addition of a control group.

## Introduction

Pseudomonas aeruginosa is a common cause of microbial keratitis that can cause a significant loss of visual acuity [1]. In contact lens users, Pseudomonas-induced keratitis can progress rapidly to corneal perforation within 24 hours [2-4]. Pharmaceutical treatments must therefore be effective at rapidly eradicating the organism. Fluoroquinolone and aminoglycoside antibiotics, including ceftazidime, gentamicin, ciprofloxacin, and combinations thereof, have been empirically shown to be effective and are the current standard treatment responses [5-6]. Intensive monotherapy is adequate if the corneal ulcer is small and peripheral with no impending perforation; but combination antibiotic therapy is recommended for more severe cases.

There have been recent reports of microbial resistance to gentamicin and ciprofloxacin in Australia, Europe, and North America [7-10]. As resistant pathogens become more common and more potent, antibiotics’ effectiveness has been conspicuously reduced. Alternative antimicrobial strategies are therefore needed. This has prompted a number of studies that have reevaluated the clinical therapeutic benefits of ancient remedies, including honey, which is known to have antimicrobial and wound-healing properties [11-15].

Tualang honey is produced by the rock bee (apis dorsata), which builds hives high up in the branches of the tualang tree (kompassia excelsa or mengaris) in Malaysia. It is commonly used by locals as both a food and as a medicinal product. It has been demonstrated to have bactericidal and bacteriostatic properties against a wide range of bacteria [16-17], and has been shown to inhibit the growth of several bacterial strains, including Streptococcus pyogenes, Staphylococcus aureus, Salmonella typhi, coagulase-negative Staphylococcus spp, Escherichia coli, Pseudomonas aeruginosa, and Acinetobacter baumanii [18]. It also has anti-inflammatory and antioxidant properties that can facilitate wound healing [15, 19-20]. Patients have also reported that they prefer the tualang honey hydrogel dressings to conventional dressings because the treatment has a soothing effect, reduces discomfort during dressing changes, and provides a long-lasting pleasant odor [21].

The potency of tualang honey against microorganisms suggests its potential as an alternative therapeutic agent for certain medical conditions, especially wound infections [15, 19]. However, no experimental in-vivo study has been published that quantifies the effectiveness of tualang honey in treating infective eye conditions. The present study thus sought to analyze the clinical and antibacterial effects of tualang honey for treating Pseudomonas-induced keratitis in rabbit eyes.

## Materials and methods

Materials and methods

We conducted a randomized control animal experiment between September 2014 and September 2016 at the Animal Research and Service Centre of the Microbiology Laboratory and Pharmacological Laboratory at the Universiti Sains Malaysia (Health Campus, Kubang Kerian, Kelantan, Malaysia). The study was approved by the research and ethical committee at the School of Medical Sciences and the Animal Ethics Committee at the Universiti Sains Malaysia Health Campus. A preliminary pilot study was conducted to verify the feasibility of the methods and clinical results.

Animals

Three rabbits were used for the preliminary pilot study and 30 rabbits were used in the main study. All specimens were New Zealand white adult rabbits aged eight to 10 months that weighed 2.5-3.0 kg and had clear corneas prior to beginning the experiment. The rabbits were maintained and handled according to ethical guidelines for the treatment of animals. They were housed individually in stainless steel cages at a controlled temperature and humidity level and had a 12-hour light/dark cycle. Food (pellets) and water were provided ad libitum. Prior to inoculation with the bacteria, the rabbits were anesthetized with ketamine hydrochloride (100 mg/ml; 35 mg/kg) and xylazine hydrochloride (20 mg/ml; 2.4 mg/kg). A drop of proparacaine hydrochloride 0.5% was then topically applied to the right eye of each rabbit. They were euthanized on day seven after injection of pseudomonas aeruginosa. During euthanasia, they were first anesthetized and were then administered a lethal overdose of pentobarbital (60 mg/ml; 125 mg/kg).

Bacterial strain and infection

Pseudomonas aeruginosa (strain 27853) were grown in tryptic soy agar (TSA) overnight at 37 °C. The overnight culture was then inoculated into fresh tryptic soy broth (TSB) (1:100) and grown at 37 °C for 18 hours. The bacteria were then serially diluted in TSB to 10,000 colony-forming units (CFU) per 1 ml. The diluted bacteria were then plated on TSA and their number was verified with a quantitative bacterial count. The right cornea of each rabbit was injected intrastromally with 1,000 CFU of Pseudomonas aeruginosa in 0.1 ml of TSB [22-24].

Treatment regime

In the preliminary study, the rabbits were injected with Pseudomonas aeruginosa and then treated with a single topical drop of saline eye drops every two hours from 24 to 48 hours after injection, and subsequently every four hours until day seven, at which point they were euthanized.

In the actual study, the rabbits were randomized into three groups of 10 rabbits each (n = 30). Group A was treated with topical gentamicin 0.3%, group B was treated with topical tualang honey 30%, and group C received both treatments. The specified treatments were administered every two hours from 24 to 48 hours after injection, and subsequently every four hours for six days. On day seven, post induction of Pseudomonas, all rabbits were euthanized and their corneas were harvested to determine the median CFU per cornea.

Clinical eye examinations

To evaluate pathological changes in the eyes, a single blinded examiner performed slit lamp examinations (SLEs) on all rabbit corneas on days one, two, three, five, and seven after the injection of Pseudomonas aeruginosa. The examinations were performed using hand-held portable slit-lamp biomicroscope, and the results were clinically recorded using an ulcer scoring system for corneal infiltrates, corneal ulcers, hypopyons, and corneal perforations (Table 1); the possible scores ranged from zero (normal) to a maximum of four [25].

**Table 1:** Slit Lamp Examination (SLE) scores Adapted from Dong et al*. *[25].

Grade	Focus of infection		
0	No focus of infection	0	
1	Corneal Infiltrate	1.25	Corneal infiltrate limited in the inoculated area
		1.50	Corneal infiltrate ≤ ½ corneal thickness
		1.75	Corneal infiltrate > ½ corneal thickness
2	Corneal Ulcer	2.25	Diameter ≤ 3mm
		2.50	> 3mm diameter < 5mm
		2.75	Diameter ≥ 5mm
3	Hypopyon	3.25	Altitude ≤ 1/3 Anterior chamber (AC)
		3.50	>1/3 AC altitude < ½ AC
		3.75	Altitude ≥ ½ AC
4	Corneal perforation	4.00	

CFU determination

On day seven, after the injection of pseudomonas aeruginosa, the rabbits were anesthetized and then euthanized. Their corneas were harvested and homogenized in sterile phosphate buffer and the homogenates were serially diluted (1:10) in fresh TSB, plated in triplicate TSA, and incubated at 37 °C overnight. The bacterial colonies were then counted to determine the median CFU per cornea for each treatment group.

Statistical analysis

The data were analyzed using repeated measures multivariate ANOVA in Statistical Package for Social Sciences (IBM, Armonk, NY). Abnormally distributed data were analyzed using the Kruskal Wallis test. Values were expressed as medians (interquartile range). We considered p values of < 0.05 to be statistically significant.

## Results

Preliminary study (Phase I)

Clinical Anti-inflammatory Effects

The mean SLE results of the preliminary study are shown in Table 2. The mean SLE scores increased from day one to day seven post bacterial injection.

**Table 2:** Mean slit lamp examination (SLE) scores over time in the preliminary study (n=3) SD = Standard deviation

Group Time	Mean SLE score (SD)				
	Day 1	Day 2	Day 3	Day 5	Day 7
Preliminary	1.67 (0.14)	2.42 (0.72)	3.00 (0.43)	3.33 (0.14)	3.58 (0.14)

**Figure 1:**
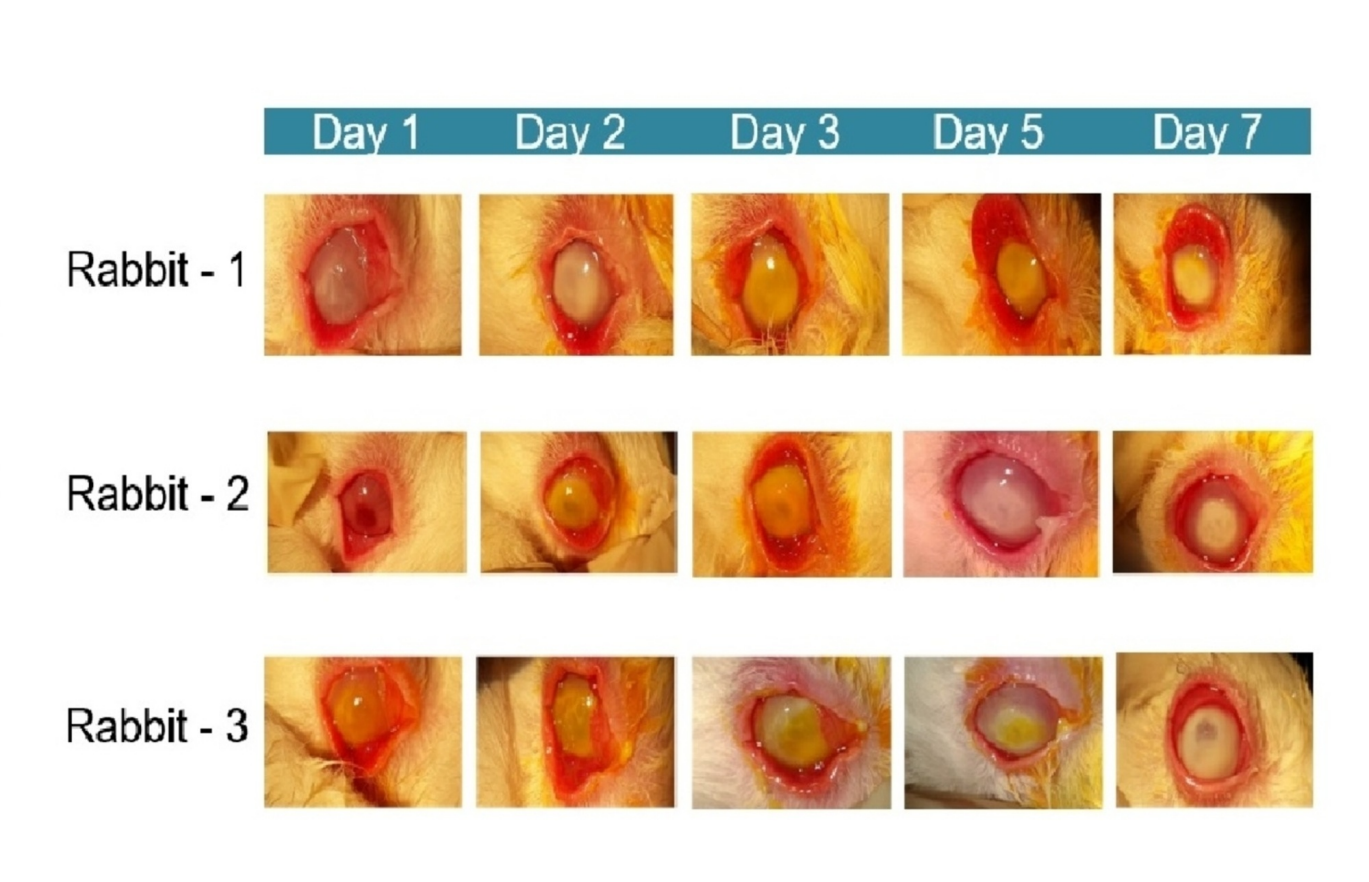
Conjunctival hyperemia and corneal edema in rabbits with Pseudomonas-induced keratitis in preliminary study

All three rabbits showed severe clinical conjunctival hyperemia and corneal edema that progressively worsened over time (Figure 1).

Table 3 shows the mean CFU count of their corneas on day seven. Although no bacterial growth was observed in rabbits one and three, this was probably due to an inaccurate calculation method of mean CFU; this method was improved for the actual study.

**Table 3:** Mean colony forming unit (CFU) of rabbits in the preliminary study on day seven after infection with Pseudomonas aeruginosa SD: Standard deviation; CFU: colony forming unit

Rabbit	CFU count
1	No growth
2	1070
3	No growth

Actual study (Phase II)

Clinical Anti-inflammatory Effects

**Figure 2:**
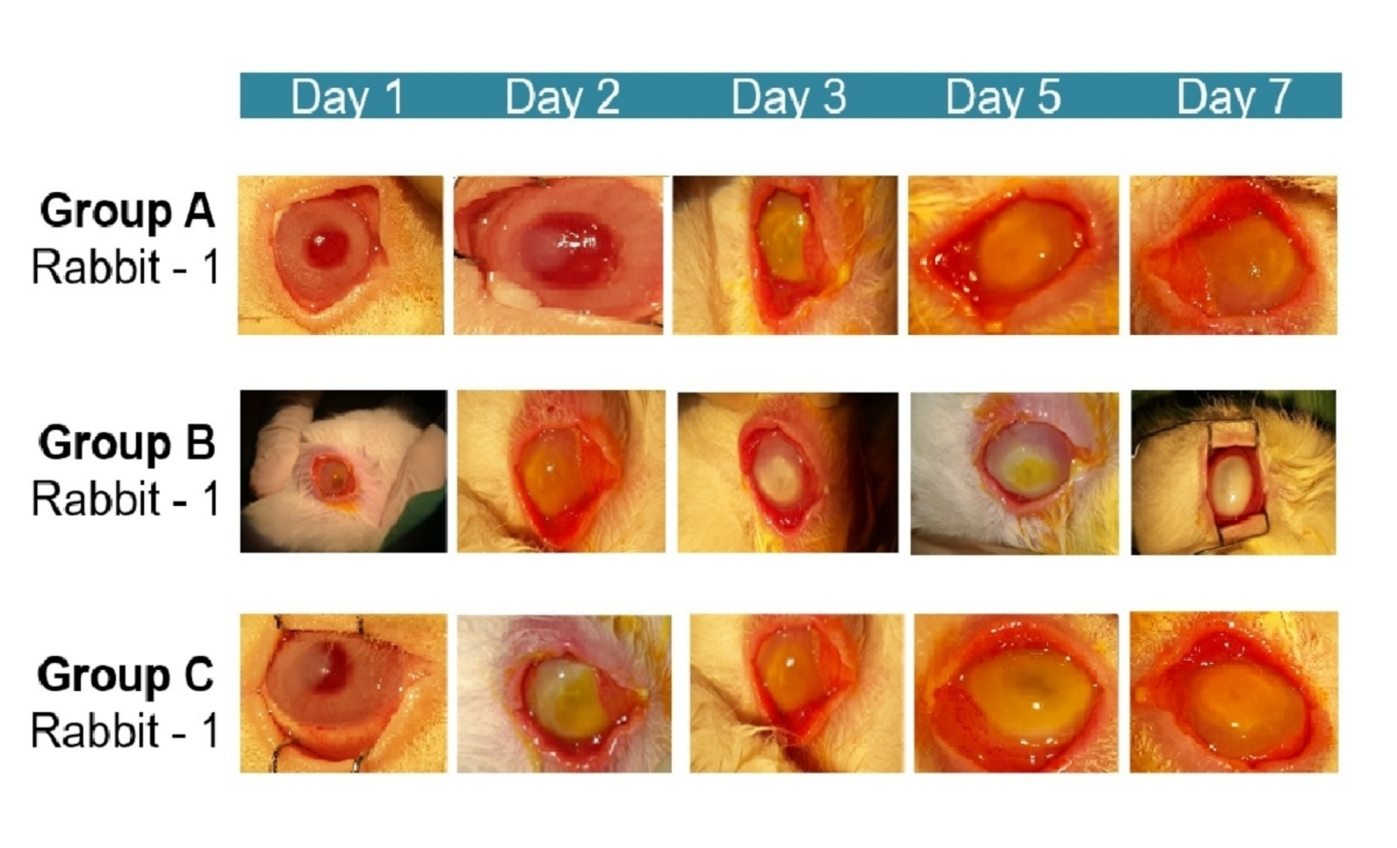
Conjunctival hyperemia and corneal edema in rabbits with Pseudomonas-induced keratitis in the actual study

In all three groups, all of the rabbits’ eyes demonstrated severe clinical inflammatory features (conjunctival hyperemia and corneal edema) on day one post infection with Pseudomonas aeruginosa. In all three groups, the conjunctival hyperemia improved progressively from day one until day seven, while the corneal edema worsened from day one until day three and then remained the same until day seven. The observed clinical features of both conjunctival hyperemia and corneal edema in all three different treatment groups are shown in Figure 2.

SLE scores

Table 4 shows the total mean SLE scores for each treatment group. The scores were almost identical between group A (gentamicin) and group C (both gentamicin and tualang honey), and there were no statistically significant differences between any of the three groups (p = 0.209).

**Table 4:** Total mean slit lamp examination (SLE) scores of each treatment group A: Gentamicin 0.3% treated group; B: Tualang honey 30% treated group; C: Combination of gentamicin 0.3% and tualang honey 30% treated groups; df: degree of freedom. Repeated measure ANOVA adjustment by Bonferroni correction, p-value <0.05 (significant)

Group	Mean difference (95% CI)	F statistic (df 1, df 2)	p value
A	2.99 (2.86, 3.11)	1.66 (2, 27)	0.209
B	2.88 (2.75, 3.01)		
C	3.04 (2.91, 3.17)		

Table 5 presents the mean SLE scores of each treatment group at different time points. The mean SLE scores in all three groups increased progressively over the study period. Notably, on day five, there was a significant difference in the mean SLE scores of groups B (tualang honey) and C (both gentamicin and tualang honey).

**Table 5:** Mean slit lamp examination (SLE) scores of each treatment group over time A: Gentamicin 0.3% treated group; B: Tualang honey 30% treated group; C: Combination of gentamicin 0.3% and tualang honey 30% treated groups. Repeated measures ANOVA within group analysis with 95% confidence interval adjustment by Bonferroni correction, p-value <0.05 (significant) with *F* statistic 1.66 (2, 27)

Group	Mean SLE score (SD)				
	Day 1	Day 2	Day 3	Day 5	Day 7
A	2.28 (0.55)	2.89 (0.16)	3.06 (0.11)	3.15 (0.12)	3.60 (0.12)
B	2.33 (0.55)	3.03 (0.13)	2.83 (0.12)	2.83 (0.11)	3.40 (0.18)
C	2.25 (0.55)	3.13 (0.13)	3.18 (0.18)	3.33 (0.11)	3.33 (0.12)
P(A) : (B)	>0.95	>0.95	0.711	0.118	0.709
P(A) : (C)	>0.95	0.510	>0.95	0.761	0.322
P(B) : (C)	>0.95	>0.95	0.131	0.008	>0.95

Antimicrobial effects

The bacterial CFU count of each treatment group on day seven post infection is shown in Table 6. Group C (both gentamicin and tualang honey) had the lowest median CFU count (48), while Group B (tualang honey) had the highest. However, the differences between the three groups were not statistically significant (p = 0.82).

**Table 6:** Median colony forming unit (CFU) of rabbits in each treatment group on day seven after infection with Pseudomonas aeruginosa A: Gentamicin 0.3% treated group; B: Tualang honey 30% treated group; C: Combination of gentamicin 0.3% and tualang honey 30% treated groups; df: degree of freedom; IQR: interquartile range. Kruskal Wallis test, p-value <0.05 (significant)

Groups	Median CFU (IQR)	Chi square statistic (df)	p- value
A	280 (899)	4.993 (2)	0.820
B	699 (835)		
C	48 (1353)		

## Discussion

Multiple studies across a variety of disciplines have reported on the uses of tualang honey [21,26-27], and more particularly on its use as a treatment for a variety of eye diseases [26,28]. Tan et al. observed its antibacterial effects against Pseudomonas aeruginosa isolates at concentrations of ≥25% [19], while Cernak et al. found that its antimicrobial properties could be used to prevent corneal scarring caused by infections [28]. Other studies have found that its antioxidant properties neutralize free radicals and gives it an anti-inflammatory effect [15-17], which can expedite treatment of blepharitis and keratitis [29].

In the present study, we found no statistically significant differences in the total mean SLE scores of the groups treated with tualang honey versus gentamicin or both (p > 0.209). The mean SLE scores in all three groups increased from day one to day seven post-infection, which suggests ongoing inflammatory processes during the early phase of infection. This is consistent with a study performed by Dong et al. that reported increasing inflammation from days four to ten in the eyes of infected rabbits treated with topical natamycin and natacyn, with subsequent inflammation regression until the end of the study on day 21 [25].

We also observed similar clinical responses to Pseudomonas-induced keratitis with all three treatment groups during the study period. These findings are consistent with Bashkaran et al, who found that the honey-treated group was as good as the conventionally treated group in terms of the healing process of an alkali-induced corneal epithelial defect in rabbits [30]. However, we were not able to find any other similar studies.

We observed no statistically significant differences in the median CFUs on day seven of our study (p >0.820). However, we found that there was a similar treatment benefit in all three groups with a reduction of median CFU on day seven. This finding is inconsistent with the study done by Nejabat et al. who found that topical 90% concentrated natural honey was equally effective as ciprofloxacin 0.3% at treating Pseudomonas-induced keratitis in rabbit eyes and that both treatments led to a significant reduction in the number of bacteria compared to controls [23].

The present study was limited by small sample size and did not include a control group due to financial constraints. Furthermore, we used tualang honey 30%, and while this is above the minimum tested bactericidal concentration of 25% for Pseudomonas aeruginosa isolates [16], it is plausible that more concentrated tualang honey might yield better results. Finally, although the rabbit intrastromal injection model is widely used and accepted as an in-vivo keratitis model for comparison to human eye infections, and while the rabbits in the present study were standardized in regard to age, gender, size, and source, the possibility always remains that the immune response observed in the rabbits here may not be generalizable to other rabbit or human populations due to genetic variations [24].

## Conclusions

Topical gentamicin, topical tualang honey, and a combination of the two all demonstrated similar clinical and antimicrobial effects in treating Pseudomonas-induced keratitis in rabbits. Additional research with larger sample sizes and the addition of a control group are warranted to further explore the antibacterial effects of tualang honey in Pseudomonas-induced keratitis.
